# Addressing the Structural Organization of Silicone Alternatives in Formulations by Molecular Dynamics Simulations and a Novel Equilibration Protocol

**DOI:** 10.3390/polym15040796

**Published:** 2023-02-04

**Authors:** Tiago Ferreira, Ana Loureiro, Jennifer Noro, Artur Cavaco-Paulo, Tarsila G. Castro

**Affiliations:** 1 CEB—Centre of Biological Engineering, University of Minho, Campus de Gualtar, 4710-057 Braga, Portugal; 2 LABBELS—Associate Laboratory, Campus de Gualtar, 4710-053 Braga, Portugal; 3 SOLFARCOS—Pharmaceutical and Cosmetic Solutions, 4710-053 Braga, Portugal

**Keywords:** silicone alternatives, branched-chain esters, molecular dynamics simulations, cosmetic formulations, emulsions, density

## Abstract

The world of cosmetics is an always-evolving field with constant updates on its formulation components. The current reality asks for an ever-increasing need for natural and sustainable replacements for synthetic compounds in all fields of modern consumer products. However, the research and development stages of finding these alternatives can be an expensive, time-consuming, and often wasteful process that turns this task into a laborious procedure. This study introduces the development of a computational methodology that will aid the research of silicone alternatives, disclosing their structural performance in a formulation. Additionally, an equilibration protocol was developed to measure the distribution and densities of these silicone alternatives to determine how they behave in relation to their counterparts, using molecular dynamics simulations. Two systems were tested, A and B, where the former is composed of one ester (Dipentaerythrityl Hexa C5 Acid Ester) and the latter by an ester combined with an alkane (Triheptanoin and C13-Isoalkane); all three molecules are commercially available and widely used. Both systems were subjected to a 3-step thermal regulation strategy. The systems went through an initial simulation at 25 °C and at 70 °C, then a temperature switch took place (25 °C « 70 °C), then a shock to 200 °C, and finally a Simulated Annealing protocol reaching 250 °C. In the end, all systems converged towards micelle-like structures. These results come to further ascertain the position of computational chemistry and Molecular Dynamics Simulations as an important part of R&D processes in modern sciences and investigation.

## 1. Introduction

Molecular Dynamics (MD) simulations have established their importance in assisting the design of new compounds or processes in the pharmaceuticals and cosmetics fields [1,2,3,4]. By modelling a system as a set of particles that interacts and behaves accordingly to classical mechanics, it is possible to have insights into several molecular processes involved in cosmetics development [5,6,7]. In the past, we have focused on hair and skin molecular structures [8,9] and on proteins and peptides related to this area [10,11] by using multiscale simulations, i.e., atomistic and coarse-grained MD. Now, we present a computational strategy to design and test the behaviour of novel ester-based formulations under different conditions.

Esters, either synthetic or from natural sources, have become hits in the manufacturing of personal care products. Medium-sized chain esters, in particular, are used as emollients in the cosmetics industry, being a perfect solution for the replacement of unwanted silicones since they match the sensorial properties of these compounds [12]. There is a plethora of emollient-esters that can be chosen according to the desired sensation, which is dependent on their chemical structure. Branched-chain esters have a good fluidity to molecular weight balance [13], and representative examples are trimethylolpropane tricaprate, neopentyl glycol diheptanoate, cetyl ethylhexanoate, ethylhexyl isononanoate, heptyl undecylenate and pentaerythrityl tetraethylhexanoate. The two esters addressed in this study are dipentaerythrityl hexa C5 acid ester and triheptanoin.

To accomplish our task, three molecules, commonly found in many commercial products and known to behave most similarly to silicones, were chosen to unravel properties such as the preferable nano-organization, interaction pattern and stability, leading to a method which can be reproduced to address many other compounds prone to have a role in formulations’ development. The molecules dipentaerythrityl hexa C5 acid ester (ERY), triheptanoin (HEP), and C13-isoalkane (ISO13) were the chosen ones. The latter two molecules are commonly found together in formulations.

The ERY is an emollient which also replaces mineral oils (petrolatum) and mimics the properties of “dimethicone 350” silicone. HEP and ISO13, when combined, act as cyclomethicone substitutes (decamethylcyclopentasiloxane, D5), also having emollient properties. Although ISO13 is a petroleum-derived mineral oil, it is highly refined, has a solid human safety record and is present in many cosmetic products because it’s very synergistic, compatible with several other ingredients, is not allergenic, and is non-reactive. All three substances, ERY, HEP, and ISO13, are rated 1 out of 10 on the EWG portal regarding environmental and health safety risks [14].

These molecules were chosen because they mimic the effect of well-known and largely used silicones. Synthetic silicones such as dimethicone and cyclomethicone are used due to their mechanical properties in promoting good texture, smoothness, and ease of spreading, but their use has drawbacks to the environment. At low molecular weight, there is the possibility of overcoming biological membranes and skin barriers, compromising safety [15]. In addition, these compounds are highly insoluble, which requires the use of surfactants and/or other co-polymers in combination, which in turn means a tremendous discharge of them in wastewater. This bioaccumulation represents, therefore, a real and even larger concern for human health and the ecosystem [16,17].

Replacing silicones in formulations with molecules with comparable properties and efficacy, but most importantly, that produce greener products, is very in line with today’s interests, as consumers are more conscious and concerned about their health, well-being, and protecting nature. But how to efficiently predict the ability of molecules to behave in a similar manner to silicones in formulations?

Silicones manufactured for cosmetics are usually fluids, and their viscosity varies consonant with molecular weight. Most of these siloxanes (−R_2_Si−O−SiR_2_−, R = organic group) form macromolecular structures large enough to be considered polymers; again, those with low molecular weight, such as the trimers, tetramers, and oligomers tend to occur as oils [18]. Siloxane molecules rotate freely around the Si-O bond, so regardless of the group attached to the silicon atom (more or less bulky), the molecule is highly flexible. Linear and cyclic (poly)siloxanes are the common forms found in cosmetics products, and these molecules are highly insoluble in both water and oil. Their large free volume, which causes wider spaces between units, and the weak interactions between the alkylated chains are responsible for the low surface tension that gives these compounds the characteristics of light feel and spreadability [19,20].

The structural and physicochemical features described above give an indication of what to look for when searching for silicone replacement by esters or esters + alkane systems. In fact, a lot of products used in the substitution of these compounds already fulfil these criteria [14]. That is, for the molecules in this study, we expect to observe both physicochemical properties and structural arrangements to be similar to silicone organizations. Therefore, the MD simulations provide a reliable sample of these distributions, revealing how the involved molecules interact and behave on a molecular scale.

For the implementation of a broad range computational protocol able to study the chosen molecules and further alternatives, we opted for the GROMACS 2021 series and the Optimized Potentials for Liquid Simulations (OPLS) force field (FF) which provides an all-atom representation of the molecules and thus offers high sensibility and resolution of these simulations.

By modelling the present silicone alternatives, we concluded that common analysis implemented to address such systems, namely, the radius of gyration, intermolecular distances, the number of contacts and the potential energies, are not the best tools to describe these systems. Following the density profile instead is a powerful route to monitor equilibration, distribution, and stability.

## 2. Methodology

### 2.1. Molecules’ Design and Parametrization

**Figure 1:**
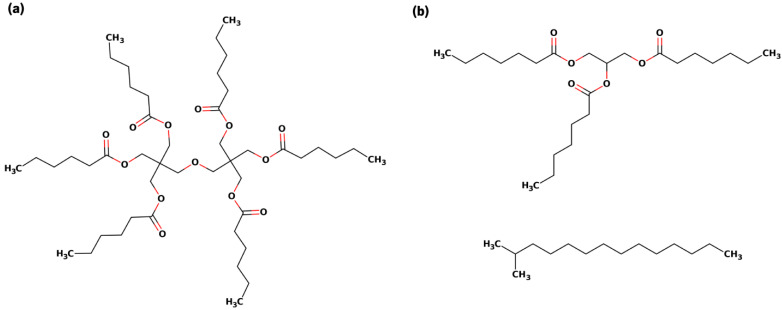
Representative 2D structures for the compounds subjected to MD simulations. In (**a**) is shown the sole compound dipentaerythrityl hexa C-5 acid ester (ERY), and in (**b**) is shown the combination between triheptanoin (above, HEP) and C-13 isoalkane (below, ISO13).

The three molecules under study, **ERY** (dipentaerythrityl hexa C5 acid ester; CAS: 67762-52-1), **HEP** (triheptanoin; CAS: 620-67-7) and **ISO13** (C13-isoalkane; CAS: 68551-20-2) were designed in 2D with the help of Marvin Sketch [21] (Figure 1) and submitted to the online platform LigParGen [22] which produced the OPLS topologies needed for simulation. All three ingredients are found in many marketable products, as summarized in the Environmental Working Group (EWG) portal [14] and highlighted in Table 1.

LigParGen performs a set of calculations that implement an algorithm refined to predict experimental parameters by using the BOSS software [23] to assign the bonded and Van der Waals parameters by analogy to the already existing atom types in the latest OPLS-AA force field. This tool will then partake in a semiempirical AM1 calculation in order to calculate and assign the charges according to 1 of 2 available C1MA-derived charge models [24].

### 2.2. Systems Design and Construction

**Figure 2:**
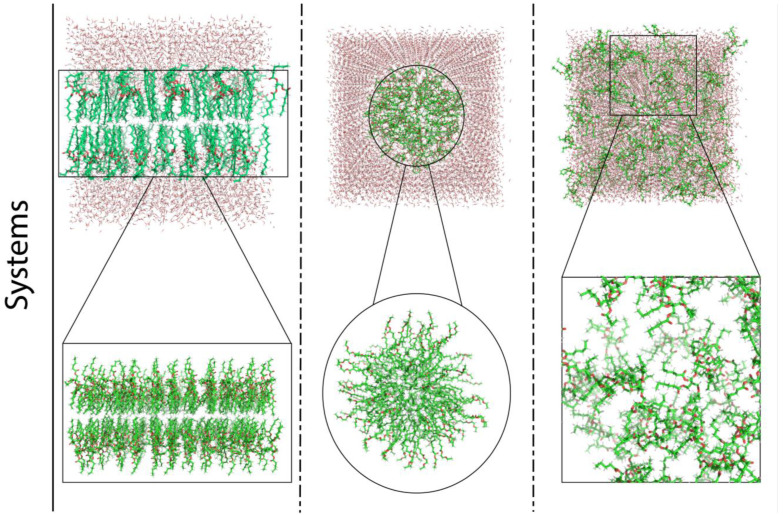
Schematisation of input structures’ design, using ERY molecules as an example. Each set of molecules will be arranged in three different systems, a bilayer-like one, a micellar one and a randomised arrangement one, respectively.

Three initial systems were designed to address the molecules’ organization, namely: bilayer-like, micelle and random-placed (Figure 2). System A is composed only of ERY molecules, whilst system B presents a combination of HEP and ISO13 in 1:1 proportion. The inputs’ designs were chosen to contemplate ordered and disordered situations; also, esters are prone to behave like fatty esters/lipids, which are commonly assembled in layered organizations [25,26].

The bilayer-like structures were achieved using the MemGen tool [27]. The bilayer-like system’s molecules were distributed with 60 Å of spacing in between, with a total amount of 100 molecules. Water molecules were added at the top and bottom extremities. In the case of the multimolecular system B (Figure 1b), HEP + ISO13, molecules were organized side-to-side in an intercalated fashion.

Micelles were put together using PACKMOL [28], with the same spacing as in the membranes beforehand (60 Å), however in a smaller quantity of molecules, 62, due to the constraints imposed by the micellar arrangement (either packing difficulties or the creation of a vacuum inside the micelle). Again, for system B, compounds were interposed in between one another.

In the third scenario, 100 ERY molecules were randomly distributed in a simulation box in the case of system A, and 50 HEP + 50 ISO13 molecules constituted system B.

All simulation boxes had approximate volumes of 125 nm^3^, and TIP3P [29] water molecules were added to solvate the systems.

### 2.3. MD Simulations Options and Analysis

All the systems were submitted to energy minimization using a maximum of 50,000 steps and the steepest descent algorithm. Initialization steps were performed using both canonical (NVT) and isothermal-isobaric (NPT) ensembles where the former maintains a constant number of particles (N), volume (V), and temperature (T) and the latter keeps the number of particles (N), pressure (P), and temperature (T) constant. The initial testing will encompass a system at room temperature, 25 °C, (298 K) and another with heating applied to 70 °C (343 K). Temperatures were thus kept constant at 298 K and 343 K, respectively, with the V-rescale algorithm [30] and the pressure regulated at 1 atm with the Parrinello–Rahman [31,32] barostat for both cases. The following coupling constants were considered: τ_T_ = 0.10 ps and τ_P_ = 2.0 ps.

Having prepared all the systems, production runs were started. For each temperature, in all three structures illustrated in Figure 2, MD simulations were performed during 100 ns using the NPT ensemble, without position restraints. Using the built-in tools provided by GROMACS 2021.2 [33], densities were calculated at 1 ns as an initial time (t_0_) and with a 25 ns time-interval afterwards.

All simulations were performed using the computational package GROMACS 2021.2 version [34,35,36], in double-precision, with the OPLS [37,38] force field. The Lennard–Jones interactions were truncated at 1.0 nm, and we used the particle mesh Ewald (PME) [39] method for electrostatic interactions, with a cut-off of 1.0 nm. Plotting of data was achieved using GNUPLOT [40].

Subsequent MD simulations take place from the initial testing at 298 K and 343 K. Thus, the 2nd round simulation consisted of submitting these systems to shock events in temperature on 2 different strategies: in the 1st, we switch the systems’ temperatures from 298 K to 343 K and the inverse, from 343 K to 298 K; the second strategy rises the temperature of both systems to 473 K (~200 °C). The 3rd round of this work will grab the layer-like systems and put them through simulated annealing until it reaches the maximum feasible temperature, at approximately 533 K (~260 °C).

## 3. Results and Discussion

After the initial steps described in the Methods section, a strategy must be placed in order to correctly assess the behaviours of these compounds in solution if the systems’ equilibration were not observed at 25° or 70° during 100 ns of simulation time. We emphasize that here ‘equilibration’ is not referring to the typical short initialization/equilibration steps performed in a simulation protocol to equilibrate temperature and pressure at desired values but rather the observation of a stable distribution of molecules in a given conformation or supramolecular structure, for a longer period of time.

**Figure 3:**
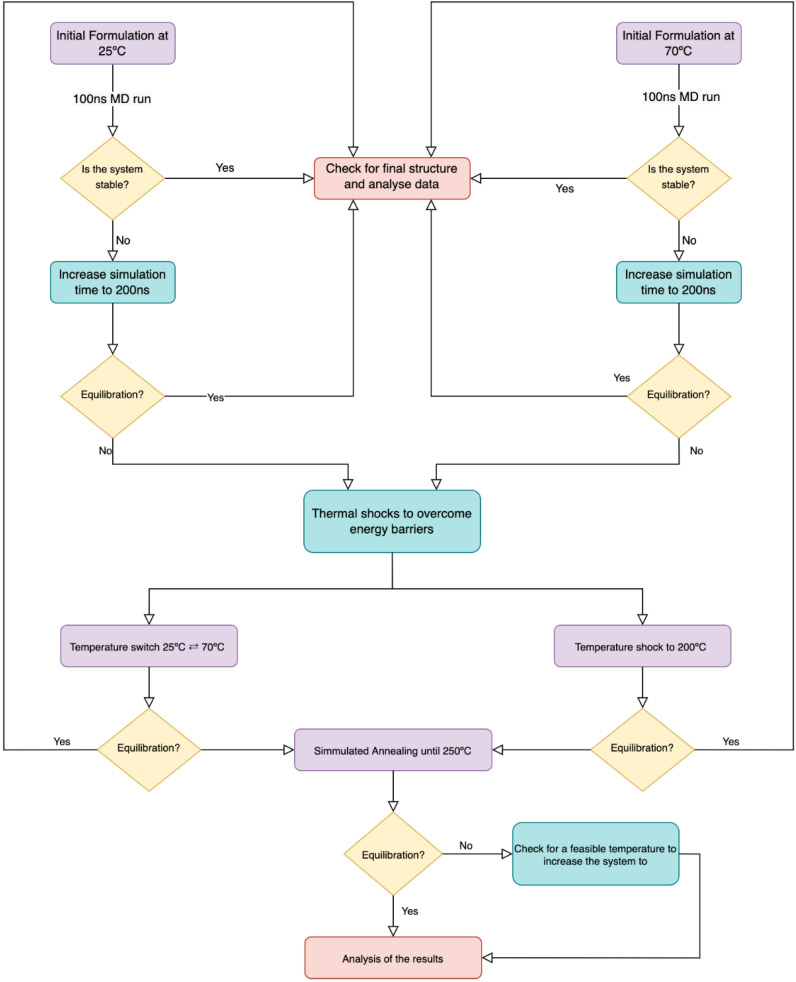
Flowchart of the simulation protocol achieved after the results were analysed and compared.

The diagram presented in Figure 3 helps to understand the rationale used to achieve systems equilibration/stabilization and to assess their preferable supramolecular structures. Initially, we look at the molecules at room temperature (25 °C) or by adding an entropic factor (70 °C) that mimics an agitation procedure. The temperature raising interferes with the internal energy of the molecules, having an impact on the molecules’ motion, which also facilitates the assembly of molecules. The results were collected in the form of particle density through time—for each 20 ns time window (up to 100 ns), then at every 50 ns (up to 200 ns)—and then analysed, with the protocol adjusted until we had achieved stability. As the flowchart suggests, strategies like increasing simulation time, thermal shocks and simulated-annealing methods were applied to fully understand our systems and are detailed in the sections below.

### 3.1. Systems at 25 °C and 70 °C

The first step, as previously introduced, was the simulations at 25 °C and at 70 °C. These temperatures were chosen based on the fact that many formulations are produced at room temperature or with an element of temperature/agitation performed on them. As such, we’ve conducted one system at 25 °C, representing room temperature, and one at 70 °C in order to represent formulation protocols that require heating.

**Figure 4:**
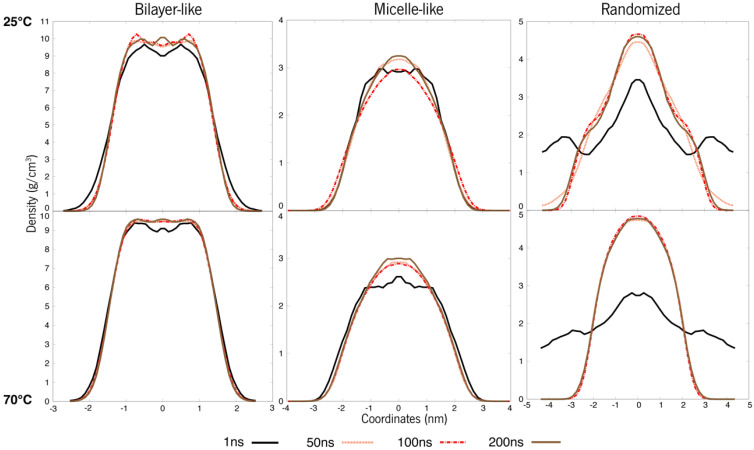
Density values of each organization of ERY (system A). The top row refers to 25 °C, whereas the bottom points to 70 °C.

As presented in Figure 4 below, it is observable that at 100 ns, these systems seemed to not be very equilibrated—more perceptible in Figure S3 with zoom-in at shorter time windows. As such, simulations were prolonged up to 200 ns until the density curves overlapped, indicating that the system had reached structural stability.

There is a notable difference among the same constructions at 25 °C and 70 °C. The curves on the bottom row are more superimposed than the ones at 25 °C, and this is indicative of equilibrium. Meaning that throughout time, the relative densities of these molecules are more or less equivalent, whereas, at 25 °C, the curves oscillate a bit more. Another important observation is the curves’ overall shape. The first system, initially a bilayer, shows a slope in the density curve at 25 °C, a feature that is typical for these layered structures and commonly observed for lipidic membranes [41]. When we look at the same system with a higher temperature (70 °C), this slope is less accentuated. This data deserves pursuing, as the system may be converging to a more “spherical” like attitude, and as such, the systems will need further experimentation. However, an overall tendency can be observed towards “micellization” in all the systems.

**Figure 5:**
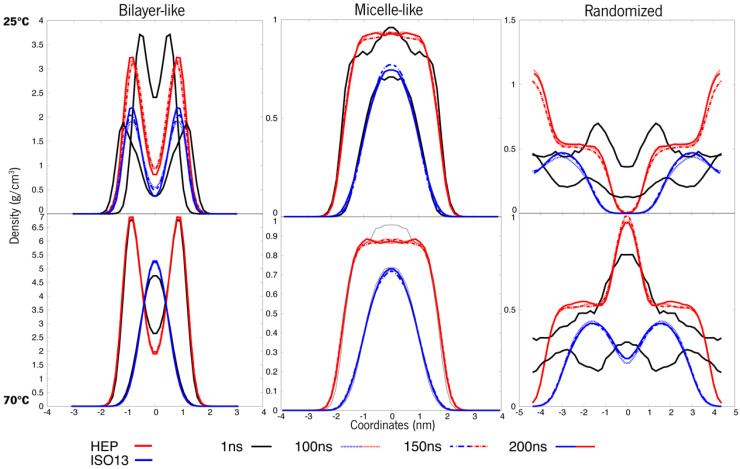
Density values of each organization of HEP + ISO13 systems (system B). The top line refers to 25 °C, whereas the bottom ones are systems at 70 °C.

Similarly, for system B, the arrangements were not completely equilibrated at 100 ns of simulation time (more perceptible in Figure S4 with zoom-in at every 20 ns), being more stable when approaching 200 ns, as seen in Figure 5. The bilayer-like system at 25 °C is still presenting unstable behaviour, and looking at its 70 °C counterpart, the inner blue line representing ISO13, is converging to a micelle-like structure first, whilst the outer ester, less hydrophobic, is still in a pattern representing a layer. These systems only appear more stable in the micellar and randomised initial structures, and this system’s molecules appear to self-assemble in an aggregate structure. At 25 °C, the superstructures remain as the initial design, with the exception of the randomised system. At 70 °C, much of the same is happening, with a noticeable more stable behaviour and a distinctive attitude in the randomised system where the roles seem to switch; when HEP seems to tend towards a micelle, ISO13 behaves in a layered-like fashion. Having that said, stability was not acquired throughout the whole 200 ns, especially in the bilayer-like organization, and as such, no major information can be interpreted from such an unstable system.

Important evidence that these systems require long simulations (around 200 ns), and a temperature component, are the other analysis performed on them. For instance, the Radius of gyration (Rg, Figures S1 and S2), Potential Energy (Figures S5 and S6) or by following the number of contacts or the intermolecular distance over time, demonstrated that these tools could erroneously indicate that structures stabilize very quickly, which is incompatible with the density distribution or the conformational snapshots over time. Literature commonly treats this kind of system through these analyses [42,43], but now we identify that the density profile might be more informative about molecules distribution, stability, and preferable arrangement.

### 3.2. Temperature-Dependent Behaviour

After having increased the time of MD run by two-fold and not having acquired stability of molecules’ distribution, in the case of the initial bilayer-like arrangement, it looks like these systems may be in a state where they’ve reached a potential energy threshold. Thus, our *in-silico* approach was to perform a thermal shock in the system in order to break that threshold and reach the state where these molecules correctly stabilize. Experimentally, the fabrication process of formulations does not account for such temperature increase as in these simulations; however, it does use efficient mechanical agitation. In an in silico context, one strategy to surpass energy barriers and sampling wide conformational arrangements is by raising the temperature, which thus changes internal energy (kinetics and potential energy) and entropy.

Importantly, some interesting tendencies observed in the bilayers’ density curves (Figure 4 and Figure 5) also indicate that the system can be trying to converge towards some other supramolecular structure. Thus, this points to a new strategy of analysing its temperature-dependent behaviour and where that leads in terms of the final structure.

#### 3.2.1. The Switch

The obvious feature of the last analysis was the clear effect of higher temperatures on the systems. A change of entropy might be the solution towards achieving stable systems, and since we can observe better stability at 70 °C, a follow-up strategy was developed with the aim of regulating the agitation of the system via two temperature-driven methods. In the first case, the systems’ temperatures are switched (25 °C <--> 70 °C), and in the second case, the systems will suffer a temperature shock up to 200 °C (473 K).

**Figure 6:**
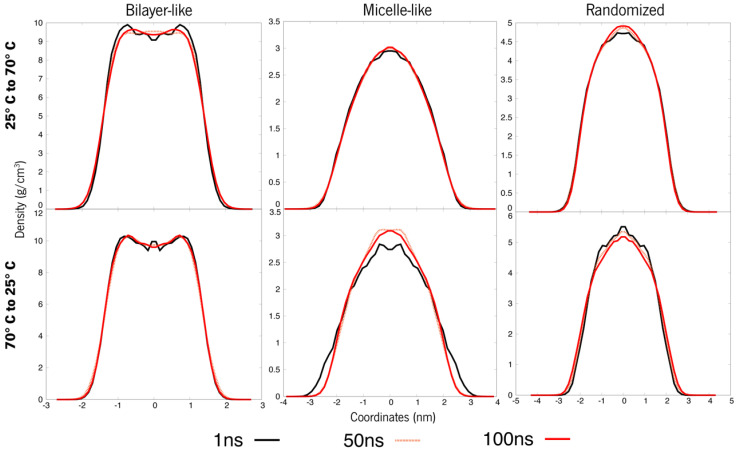
Density values of each organization for system A. The top line refers to 25–70°C, whereas the bottom ones are organizations at 70 °C turned to 25 °C.

A clear tendency towards a more micellar arrangement takes shape for system A, rising from 25° to 70 °C (Figure 6). Although these systems are now apparently stable, the bilayers keep that typical slope, but this same depression is less accentuated. Thus, adding more agitation might bring the system to further conformational changes. When systems change from 70° to 25 °C, no remarkable differences are observed, except for the bilayer-like arrangement, which displays the slope more stressed again.

Clear changes in the density curves of the systems that were heated can be observed at the level of proximity amongst them, indicating closer density values of these molecules throughout the simulation and thus showing that increasing the temperature promotes stability. Moreover, the bilayer-like system also shows fewer fluctuations in densities through its coordinates, another indicator of increased uniformity happening throughout the molecules in space. On the other hand, when this system is cooled, some previously unobserved variations now appear. However, these are small changes, which also points towards the idea that cooling down a system will have little impact when compared to the temperature increase. As already mentioned, the bilayer-like systems are more impacted by this cooling, however, as the density curves clearly show a tendency to a stable layer, with a clearly increased bulge in the density curves.

**Figure 7:**
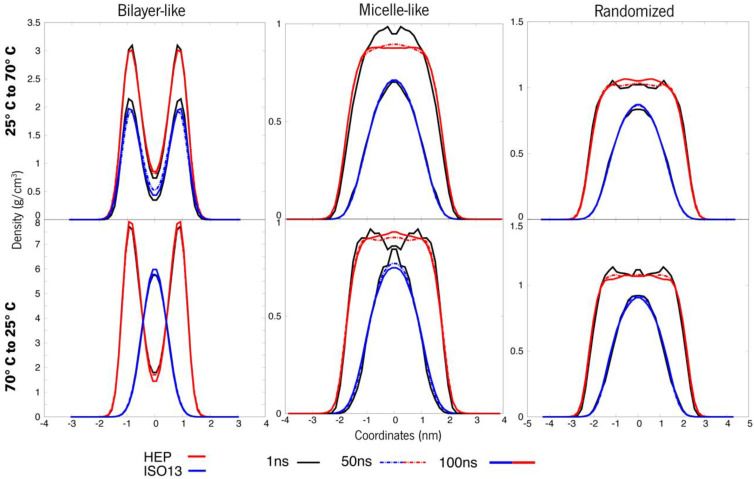
Density values of each organization for system B. The top line refers to 25–70 °C, whereas the bottom ones are systems at 70 °C turned to 25 °C.

Looking now at system B under the same strategy, its organizations, when heated, reveal now stable and overlapped curves where they previously weren’t (Figure 5 and Figure 7). Contrarily, the micelles where the temperature was lowered to 25 °C are now showing some fluctuations. The same tendency towards micelle formation appears as before, although the bilayer-like systems keep within their threshold. The comparative analysis of the previous 200 ns simulation without a temperature-driven strategy shows better stability in organizations that went through heating and a better reaction to cooling than what was observed in System A. In the case of the layered systems, whereas heating provided lower densities and, thus, a more compacted structure, cooling allowed the system to have some relaxation. This phenomenon will also cause the system to remain in two phases, where ISO13 appears to have a different organizational pattern from HEP. Altogether indicates that heating the system is more beneficial towards not only stability but system compactness and inducing transitions in the overall superstructure of the molecules.

#### 3.2.2. The Shock

The second strategy for thermal shock was to increase the temperature to 200 °C. These systems were shocked according to their initial temperatures, so we will have 25–200 °C and 70–200° C. This high temperature is expected to promote considerable changes in the most trick organizations, such as the bilayer-like, and an effect on the packing and compactness of all systems.

**Figure 8:**
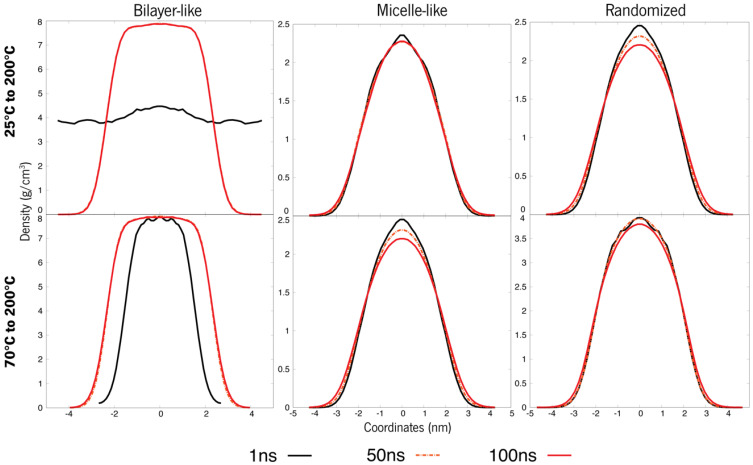
Density values of each system A. The top line refers to 25–200 °C, whereas the bottom ones are systems at an initial 70 °C that were shot up to 200 °C.

**Figure 9:**
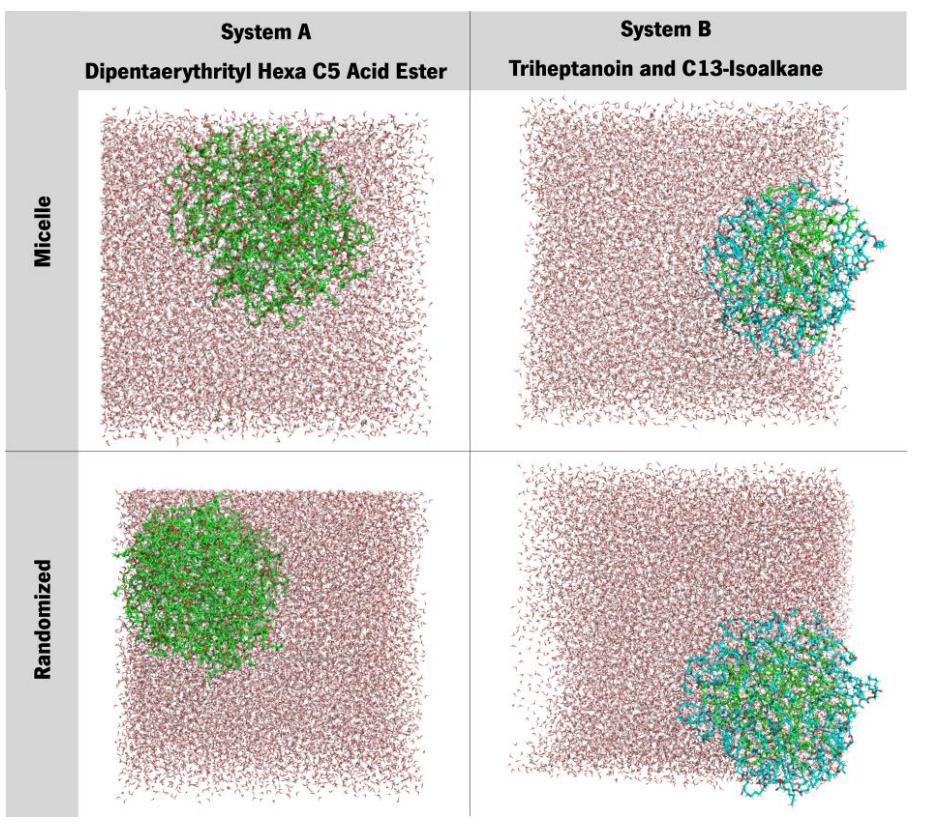
Snapshots of the last frame of systems A and B at 200 °C (from 70 °C) both in initial micellar and randomised assortments.

Analysis of Figure 8 agrees with the previously stated, as it displays a stable system on all 3 structural case studies, showing that these systems form micellar-like arrangements under temperature-induced stress. The bilayer-like arrangement, both starting at 25 °C or 70 °C, present very similar attitudes, and almost all the density values across all the tested systems coincide over time, while for micelle-like, the shock 25° to 200 °C induces a quicker stabilisation, with curves overlapping first. In particular, for the randomised systems, the molecules will assemble randomly and in different manners as the level of agitation is different at each temperature, and the visual inspections confirm the assumption that entanglement is observed rather than a well-ordered micelle organization (Figure 9). Another conclusion regards the density values, which decrease in all cases, indicating that the higher temperature does not weaken the interaction but, in turn, promotes a more stable packing.

**Figure 10:**
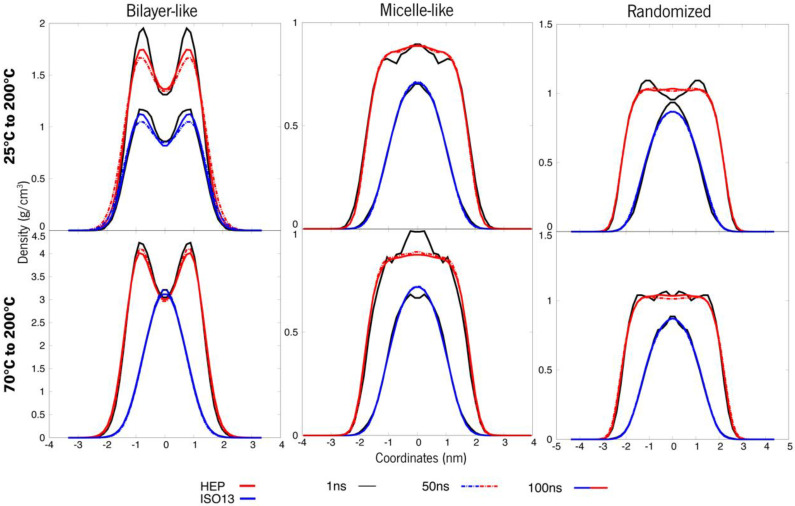
Density values of each system B organization. The top line refers to 25–200 °C shocks, whereas, at the bottom, systems at 70 °C were raised to 200 °C.

On the other hand, for system B (Figure 10) it was observed a persistent behaviour in the case of membrane organization (bilayer), as the ester remains in a layered fashion and makes difficult the transition to a micelle. The two other organizations (micelle and randomised) revealed similar behaviour between themselves. All the densities vary by a few decimals, and the density curves for both compounds in the systems are similar and resemble a micellar pattern. Another noteworthy observation is that in the randomised systems, a small compacting occurred. When compared to previous observations in Figure 5, the peak densities of these systems also lowered, and the dipped curves seem to be shrinking. Also, there is a clear separation occurring between molecules, more accentuated at the initial 25 °C, but also in the system at an initial 70 °C. This latter system also presents an increase in the lower density at the bulge on HEP, which indicates a possible tendency towards a rearrangement of the overall distribution of these molecules. Figure 9 gives a visual insight into the preferable supramolecular organization for systems submitted to a 70° to 200 °C shock.

The snapshots reveal that both systems, the one that starts as a micelle and the other that starts with the molecules randomly placed, converge to a micelle-like form, presenting an entanglement of chains, which is also typical for silicone (polydimethylsiloxane) molecules [44]. There is very little information regarding the supramolecular organization of branched esters or cosmetics’ silicones in the literature [45]. Therefore, we are shedding light on this field, bringing the contribution of molecular simulations. Experimentally, these molecules were tested by us in combination with other formulation components (data not published); hence, it is difficult to compare with other data.

### 3.3. Simulated Annealing

Simulated annealing is a heuristic optimization problem-solving strategy. It is not only the most common heuristic method for various optimization problems but also a robust technique that makes it possible to perform MD simulations by applying discrete temperature increments along a given timeframe. This translates to essentially performing a simulation run which applies gradual, slow increases of temperature with time, raising it to an otherwise unfeasible temperature. This method is very robust for conformational search and has been used in biomolecules before [46,47]. The next strategy would be to apply this technique for another 100 ns. This run will be achieved by increasing the temperature ideally to 300 °C (573 K). Through trial and error, we found out that the systems were not viable after about 250 °C, so that was taken as the maximum temperature, and the systems were then run. Annealing properties were 10 °C heating at every 20 ns, approximately. These steps were only employed for the still unstable bilayer-like structures.

**Figure 11:**
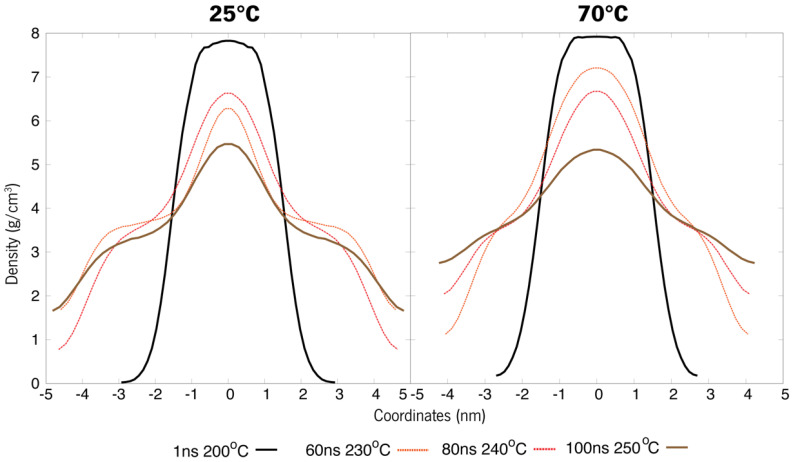
System A refers to a single compound (ERY) simulated annealing of the bilayer-like structures at both 25 °C and 70 °C initial temperatures (t0).

**Figure 12:**
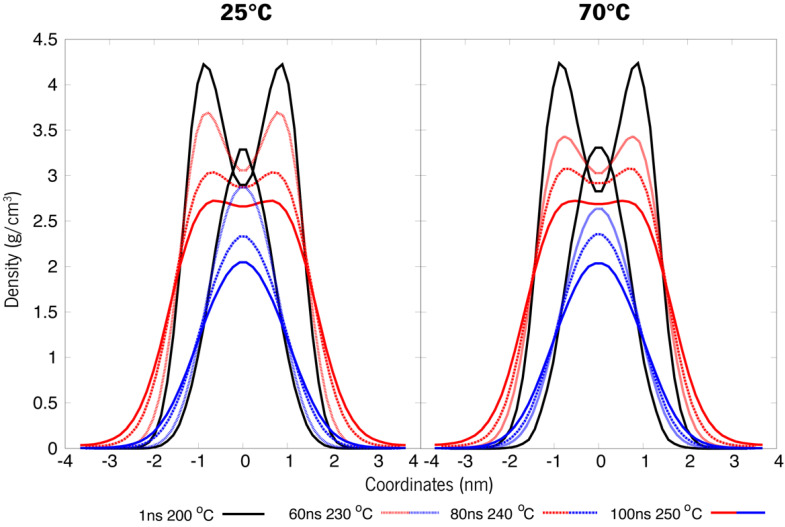
System B refers to a double compound (HEP + ISO13) simulated annealing of the bilayer-like structures at both 25 °C and 70 °C initial temperatures (t0).

**Figure 13:**
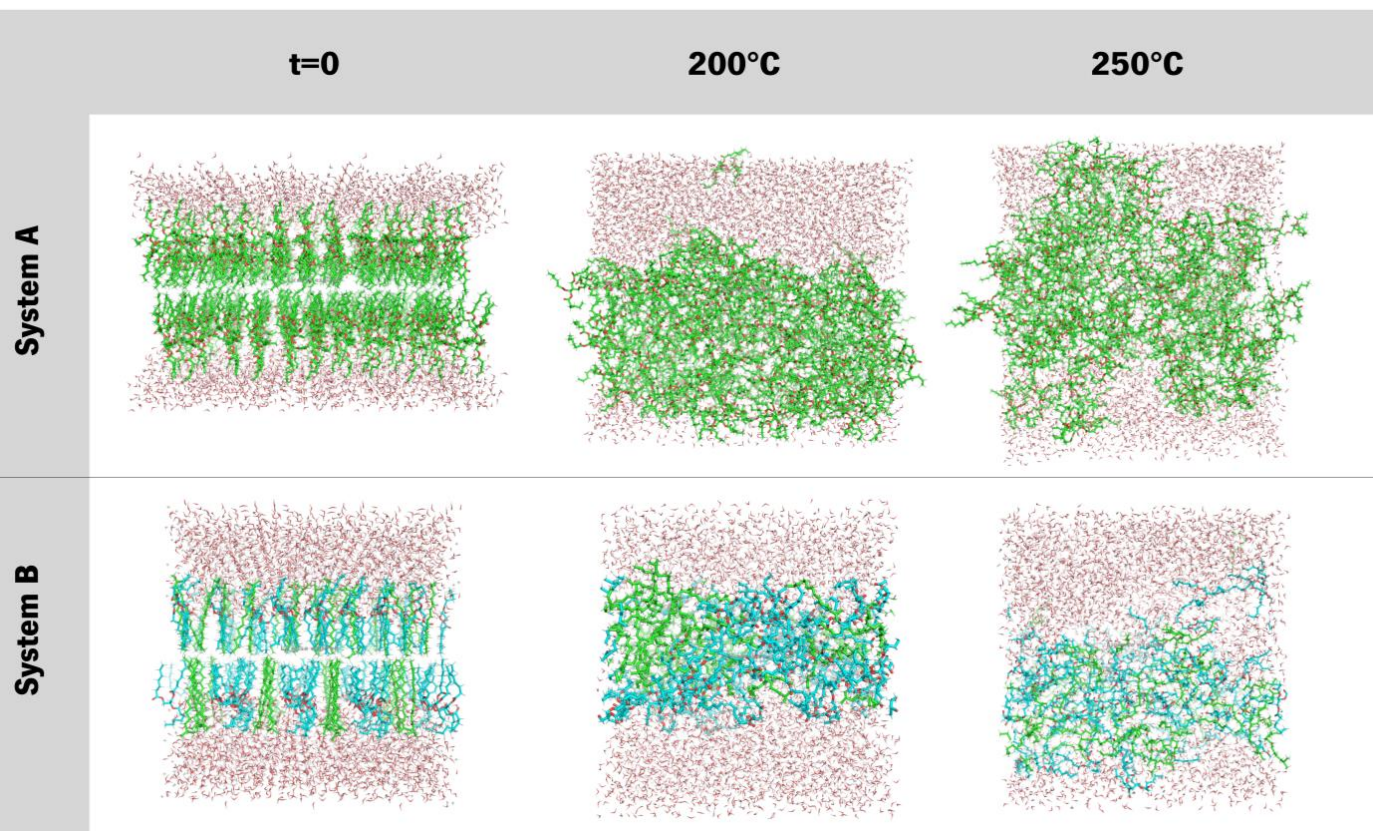
Layered systems at the three main stages of simulation, from right to left, systems at initial input (t = 0; 70 °C), 200 °C and 250 °C, respectively.

Under temperature-induced stress, all case studies display a transitional behaviour towards density patterns resembling those of micellar arrangements (Figure 11 and Figure 12). Although these new apparently micellar systems remain fluctuant in their respective densities through time, a clear-cut change in all curves indicates a change in the behaviour of these molecules, a feat also proven by Figure 13. Although system A virtually remained under the same organization as in Figure 8, these systems are now much less dense than before (Figure 11). System B (Figure 12), under further heating by annealing, also showed a destabilization of its previous states by their overall densities now increasing in comparison to Figure 9, a feat that may be explained by the breach of the strong bilayer-like arrangement and its molecules dispersing along the box. Another piece of data that can lead to this analysis is that after an initial relaxation when nearing 100 ns, densities get back to similar values as previously reported. These results will help further the research into new formulations, helping to understand the steps needed in order to study and evaluate the behaviour of de novo compounds in aqueous solutions. Additionally, these results can help to design a strategy for the production of new formulations and study the compatibility between its ingredients.

Figure 13 presents the temperature-evolution of systems A and B, from 70 °C, which acquire a random organization at 200 °C and 250 °C but display some similarities to a worm-like micelle conformation as they point the oxygen atoms towards the surrounding water.

## 4. Conclusions

The main objective of this work was to simulate the behaviour of commercial molecules commonly used in the cosmetics field and implement a computational strategy to address cosmetic formulations unravelling molecules’ distribution, supramolecular arrangements, and interactions.

Esters and alkanes, such as the ones tested herein, are mostly found arranged together in micelles, bilayers or liposome-like vesicles that are essentially bilayer-like structures. The modelling systems were split into three different simulation input structures: a bilayer-like structure, a micellar arrangement of molecules, and a self-assembly system. The latter is an ambiguous self-aggregation system that should, in theory, indicate the preferential aggregation pattern of these molecules. From an initial testing simulation phase, the results showed an overall instability. The increase in simulation time and temperature-driven shock strategies, which ranged from a temperature switch to an abrupt increase to 473 K to an annealing simulation phase, demonstrated that all systems form similar superstructures, comprising worm-like micellar forms in all systems.

These simulations have shown us that natural silicone alternatives, being employed in today’s markets, do, in fact, behave in a similar way as their counterparts [48,49]. These simulation processes can be optimized and performed with novel compounds, providing manufacturers within the cosmetics field with a wide spectrum of viable alternatives that would help replace not only silicones but surfactants, emollients, and other potentially harmful synthetic chemicals in formulations. Thus, these results not only provide important insight into the physical and chemical behaviour of sustainable silicone alternatives in solution but also pave the way for further testing smaller molecules in a complex system in a cost-effective way.

This work’s impact was aimed at proposing a reproducible protocol to study similar compounds for similar applications. In fact, it was observed that the increase in temperature mimics the agitation process used to homogenise/emulsify experimentally. This computational induction helps overcome energy barriers and induce a stable distribution of molecules in a particular supramolecular organization. Furthermore, a slow increase of temperature/agitation through time was shown to influence an otherwise unstable system to breach the potential energy threshold created by the input organizational features of the molecules.

## Figures and Tables

**Table 1:** Marketable products with the ingredients under study, according to the EWG portal.

	ERY	HEP	ISO13
facial moisturizer and treatment	1	122	9
moisturizer	-	37	3
serums & essences	1	5	27
conditioner	1	2	7
hair styling aide	1	16	3
hair treatment/serum	-	20	7
anti-ageing	-	16	1

## Data Availability

Simulations files are available upon request to the corresponding authors.

## Supplementary Materials

The following supporting information can be downloaded at: https://www.mdpi.com/article/10.3390/polym15040796/s1, Figure S1: Radius of Gyration regarding the system A, both in 25 °C (purple) and 70 °C (green), supporting the data in Figure 4. In the second row an amplified version is presented to better understand how little time it takes for the system to appear to stabilize; Figure S2: Radius of Gyration regarding the system B, both in 25 °C (purple) and 70 °C (green), supporting the data in Figure 5. In the second row an amplified version is presented to better understand how little time it takes for the system to appear to stabilize; Figure S3: Particle densities at a 5, and then 20 ns interval regarding system A. The data shown here supports Figure 4 in our work, further enhancing the irregularities in the density profile of the components throughout time in both 25 °C (top row) and 70 °C (bottom row); Figure S4: Particle densities at a 5, and then 20 ns interval regarding system B. The data shown here supports Figure 5 in our work, further enhancing the irregularities in the density profile of the components throughout time in both 25 °C (top row) and 70 °C (bottom row); Figure S5: Potential energies of system A at both 25 °C, in red, and 70 °C in green with respective running averages. The figure herein serves as support to Figure 4 in the manuscript, yet another representation of how such measurements can represent the apparent stability of these systems under study when it is not the case; Figure S6: Potential energies of system B at both 25 °C, in red, and 70 °C in green with respective running averages. The figure herein serves as support to Figure 5 in the manuscript, and like the latter image, another case where apparent, yet possibly misleading stability is shown.
