# Supplementary material for: Addressing the Structural Organization of Silicone Alternatives in Formulations by Molecular Dynamics Simulations and a Novel Equilibration Protocol
Source: Polymers (Basel). 2023 Feb 4;15(4):796. doi: 10.3390/polym15040796 (PMC9967600; doi:10.3390/polym15040796)
Supplement: Supplementary file 1 [file polymers-15-00796-s001.zip › polymers-2121007-supplementary.pdf]

Addressing the Structural Organization of Silicone Alternatives in Formulations by Molecular Dynamics
Simulations and a Novel Equilibration Protocol

Tiago Ferreira [1,2,3], Ana Loureiro [1,2,3], Jennifer Noro [1,2,3], Artur Cavaco-Paulo [1,2,3,*] and Tarsila G. Castro [1,2,*]

[1] CEB—Centre of Biological Engineering, University of Minho, Campus de Gualtar, 4710-057 Braga, Portugal
[2] LABBELS—Associate Laboratory, Campus de Gualtar, 4710-053 Braga, Portugal
[3] SOLFARCOS—Pharmaceutical and Cosmetic Solutions, 4710-053 Braga, Portugal
* Correspondence: artur@deb.uminho.pt (A.C.-P.); castro.tarsila@ceb.uminho.pt (T.G.C.)

Supporting Information

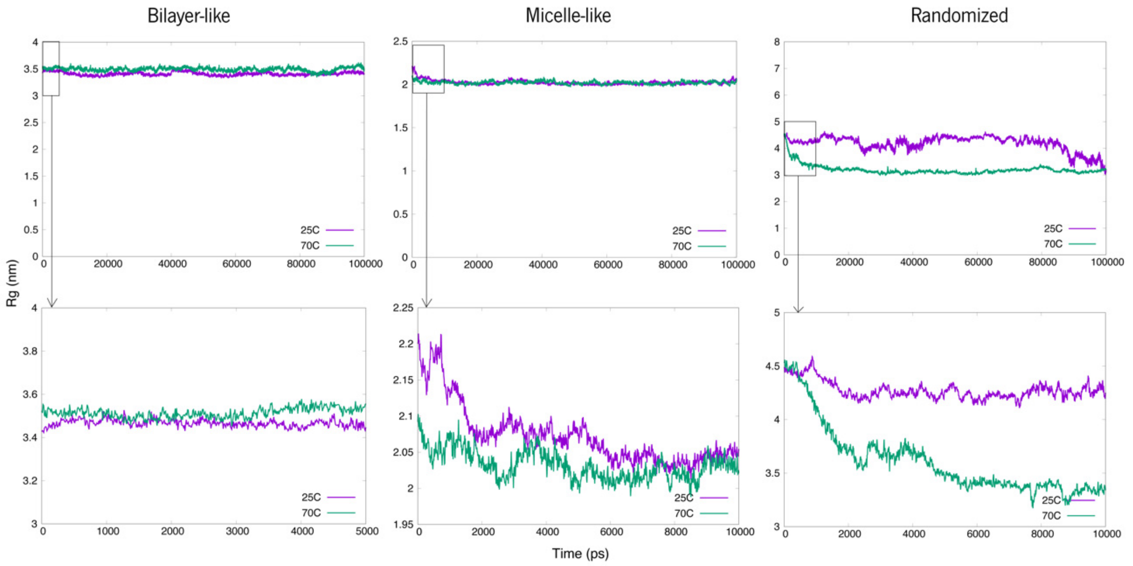

Figure S1. Radius of Gyration regarding the system A, both in 25°C (purple) and 70°C (green), supporting the data in Figure 4. In the second row an amplified version is presented to better understand how little time it takes for the system to appear to stabilize.

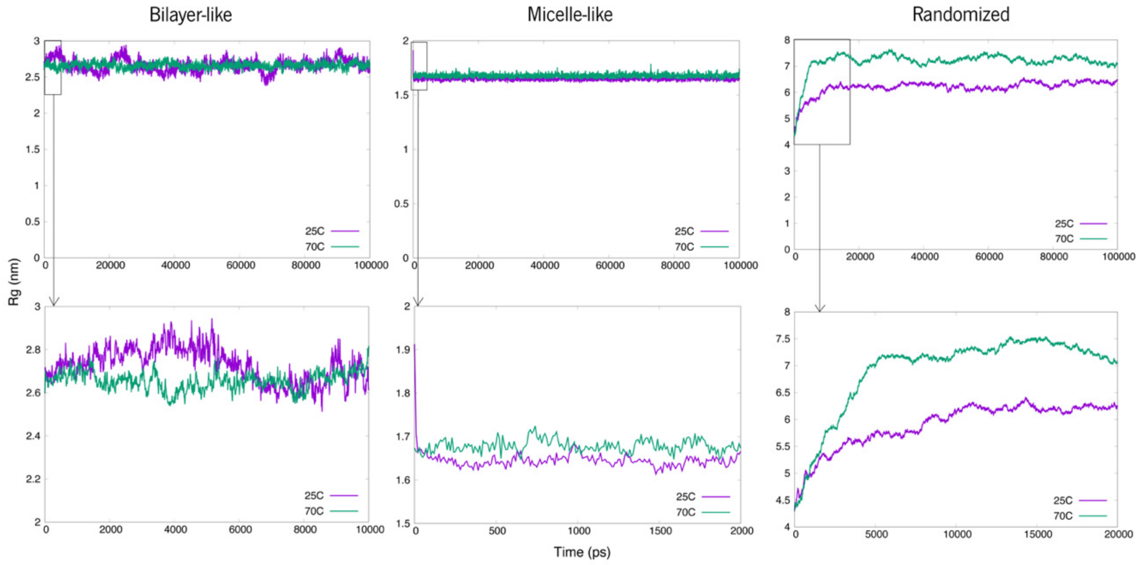

Figure S2. Radius of Gyration regarding the system B, both in 25°C (purple) and 70°C (green), supporting the data in Figure 5. In the second row an amplified version is presented to better understand how little time it takes for the system to appear to stabilize

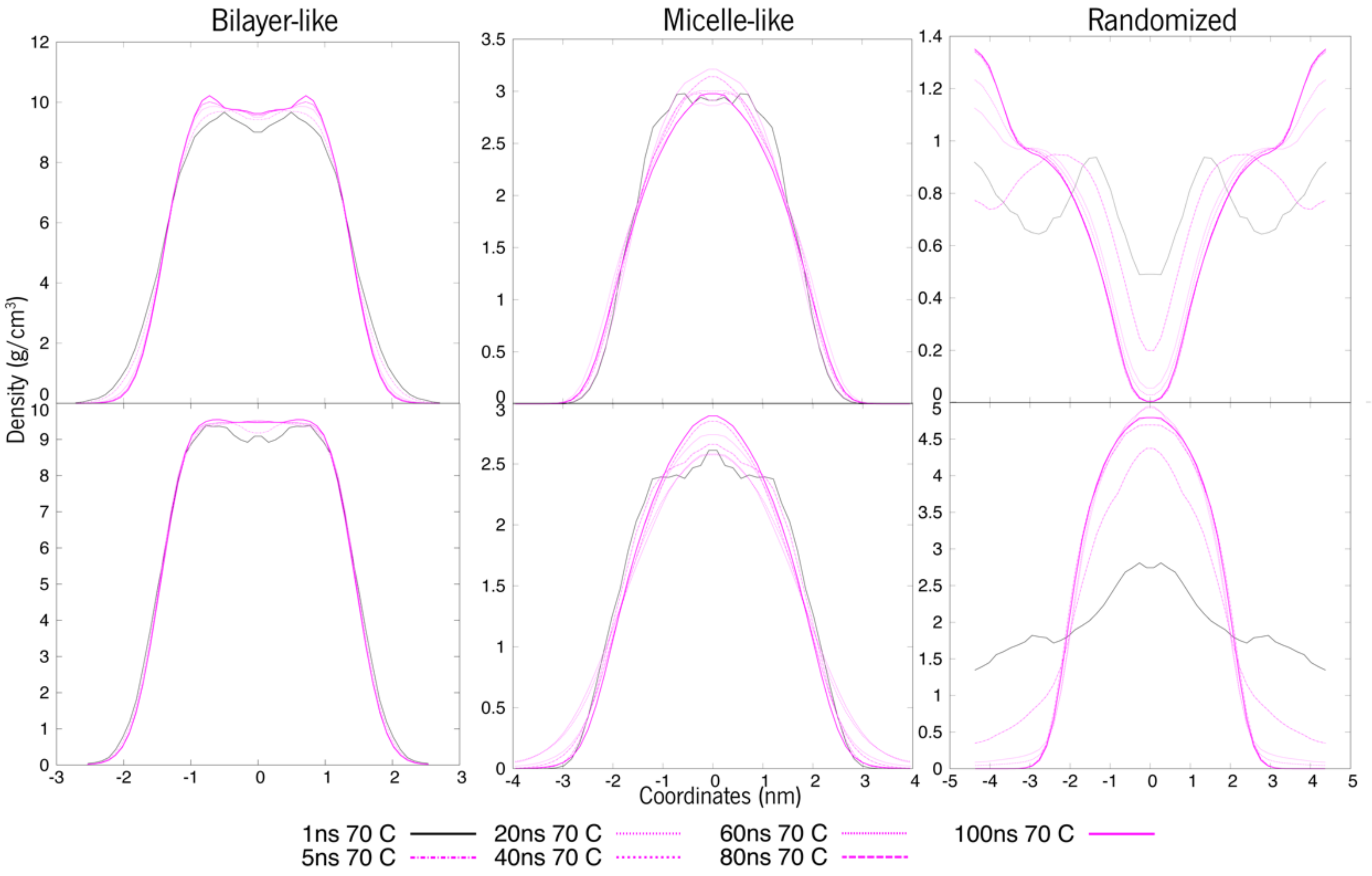

Figure S3. Particle densities at a 5, and then 20 ns interval regarding system A. The data shown here supports Figure 4 in our work, further enhancing the irregularities in the density profile of the components throughout time in both 25° C (top row) and 70° C (bottom row).

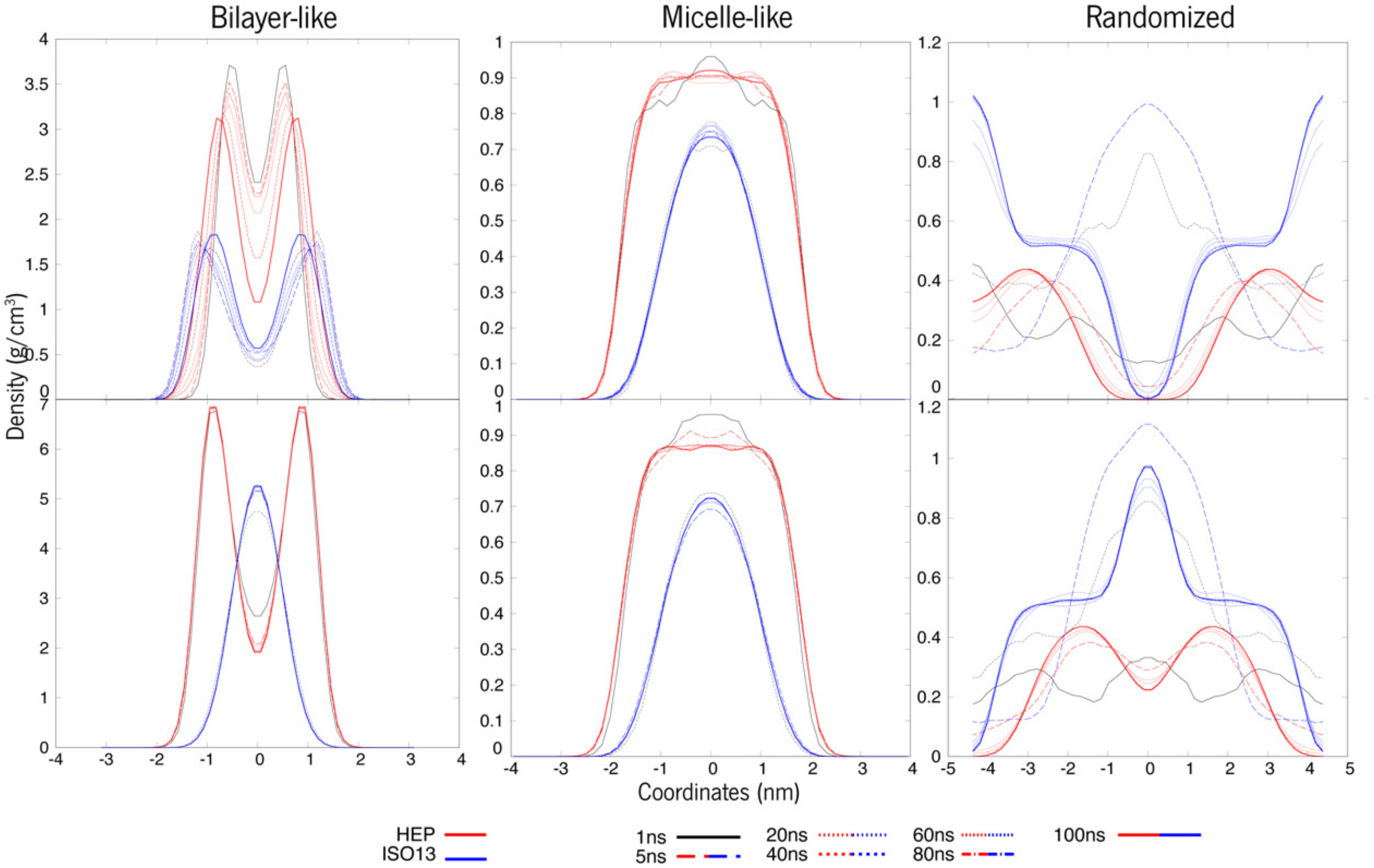

Figure S4. Particle densities at a 5, and then 20 ns interval regarding system B. The data shown here supports Figure 5 in our work, further enhancing the irregularities in the density profile of the components throughout time in both 25° C (top row) and 70° C (bottom row).

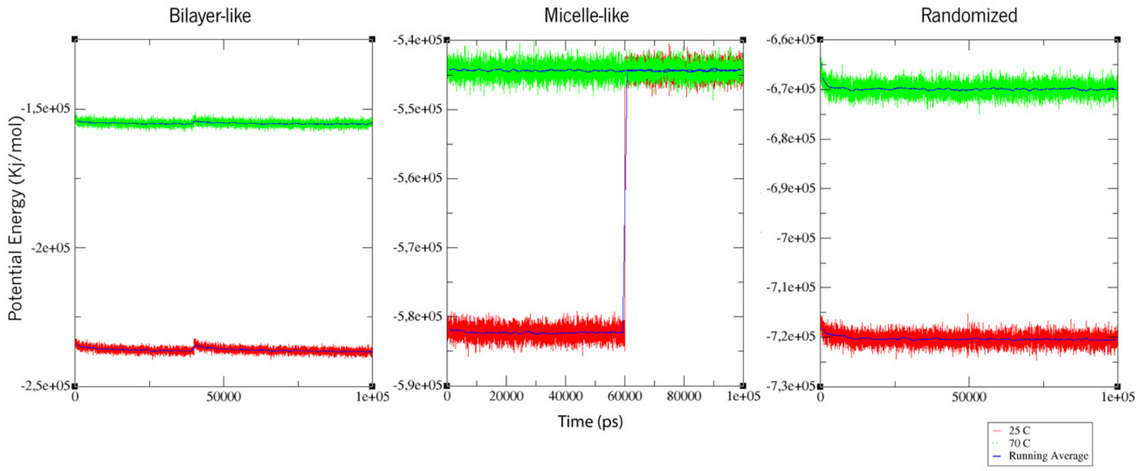

Figure S5. Potential energies of system A at both 25 °C, in red, and 70 °C in green with respective running averages. The figure herein serves as support to Figure 4 in the manuscript, yet another representation of how such measurements can represent the apparent stability of these systems under study when it is not the case.

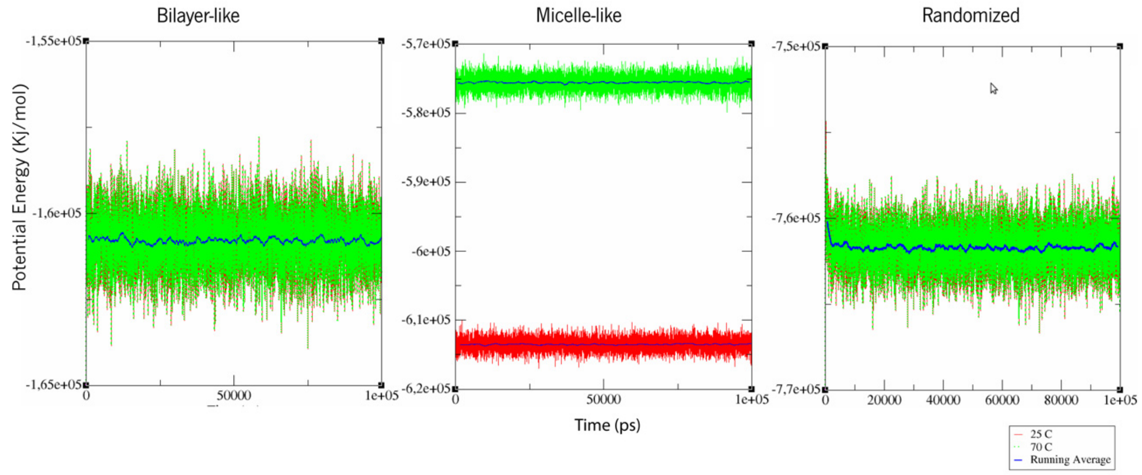

Figure S6. Potential energies of system B at both 25 °C, in red, and 70 °C in green with respective running averages. The figure herein serves as support to Figure 5 in the manuscript, and like the latter image, another case where apparent, yet possibly misleading stability is shown.
